# The use of hypnotherapy as treatment for functional stroke: A case series from a single center in the UK

**DOI:** 10.1177/1747493021995590

**Published:** 2021-02-27

**Authors:** Ranjan Sanyal, Marko Raseta, Indira Natarajan, Christine Roffe

**Affiliations:** 1 Neurosciences, Royal Stoke University Hospital, Stoke-on Trent, UK; 2 Statistics and Mathematical Modelling, Department of Molecular Genetics, Erasmus MC, Rotterdam, the Netherlands; 3 Faculty of Medicine and Health Sciences, Keele University, Staffordshire, UK

**Keywords:** Hypnotherapy, functional stroke, cost-effective, safe intervention

## Abstract

**Background:**

Functional neurological disorder is defined by symptoms not explained by the current model of disease and its pathophysiology. It is found in 8.4% of patients presenting as acute stroke. Treatment is difficult and recurrence rates are high. We introduced hypnotherapy as a therapeutic option in addition to standard stroke unit care.

**Methods:**

This is an observational study of successive patients with functional neurological disorder presenting as acute stroke treated with hypnotherapy between 1 April 2014 and 1 February 2018. The diagnosis of functional neurological disorder was confirmed by clinical examination and computed tomography/magnetic resonance imaging. Hypnosis was delivered by a hypnotherapy trained stroke physician using imagery for induction. A positive response was defined as a National Institutes of Health Stroke score reduction to 0 or by ≥4 points posthypnotherapy. Costs were calculated as therapist time and benefits as reduction in disability/bed days.

**Results:**

Sixty-eight patients (mean age 36.4 years, 52 (76%) females, mean baseline National Institutes of Health Stroke 5.0 (range 1–9)) were included. Two patients (3%) could not be hypnotized. Fifty-eight 58 (85%) responded, 47 (81%) required one treatment session, while 19% needed up to three sessions for symptomatic improvement. No adverse events were observed. Disability (modified Rankin Scale) reduced from a mean of 2.3 to 0.5 resulting in an average cost saving of £1,658 per patient. Most (n = 50, 86%) remained well without recurrence at six-month follow-up.

**Conclusions:**

In this case series, hypnotherapy was associated with rapid and sustained recovery of symptoms. A prospective randomized controlled study is required to confirm the findings and establish generalizability of the results.

## Introduction

Functional neurological disorder (FND) is a term used to describe a condition where the symptoms are not explained by the current disease model and its pathophysiology. Studies from the UK and continental Europe established that one third of the new patients seen in neurology clinics have symptoms that are only partially explained by organic disease.^1–4^ In a study of all patients presenting with acute stroke to a hyperacute stroke unit, 8.4% were found to have FNDs. Health and societal costs of FNDs were estimated to be more than £11 million per year.^5,6^ The Department of Health England estimates that medically unexplained symptoms cost the broader economy £17.6 billion each year with direct costs to the National Health Service of £3.1 billion, £5.2 billion in productivity loss, and £9.3 billion attributed to reduced quality of life.^7^ Hence, FND is a major problem facing health and social services resulting in loss of productivity, poor quality of life, and a substantial economic burden. Treatment approaches vary. NHS Scotland recommends a stepped care model starting with diagnosis and explanation, followed by brief interventions such as cognitive behavior therapy and self-help, where needed, and a multidisciplinary approach for refractory cases.^5^ A recent review of the management of FNDs in the *American Journal of Psychiatry* suggests that FND does not fit neatly into either the specialty of psychiatry or neurology and should therefore be addressed by an interdisciplinary approach with collaboration of neurologists and psychiatrists making use of a wide range of treatment approaches including antidepressants, psychotherapy, physical therapy, cognitive behavior therapy, and hypnosis.^6^

Hypnosis is a temporary condition of altered attention in the subject which may be induced by another person and in which a variety of phenomena may appear spontaneously or in response to verbal or other stimuli. These phenomena include alterations in consciousness and memory, increased susceptibility to suggestion, and the production in the subject of responses and ideas unfamiliar to them in their usual state of mind. Furthermore, phenomena such as anaesthesia, paralysis and rigidity of muscles, and vasomotor changes can be produced and removed in the hypnotic state.^8^ To date, only two randomized controlled trials of hypnosis in the treatment of functional symptoms (conversion disorder and somatoform disorder) have been reported.^9,10^ Both included patients with chronic symptoms. One, comparing a comprehensive treatment program with and without hypnosis, found no additional benefit,^9^ and the other, comparing hypnosis against waiting list controls, showed better recovery with hypnosis.^10^

There is no evidence-based effective therapy for patients presenting acutely with functional stroke symptoms. Hypnosis has been used to treat functional neurological symptoms in a range of neurological presentations. We introduced hypnotherapy as an additional treatment option for patients presenting with functional symptoms^11^ mimicking acute stroke. A pathway was developed and approved by clinical governance, which included an audit of response rates and complications. In this paper, we report the outcome of the first 68 cases treated.

## Methods

### Design

This study is a review of the outcomes and complications of a hypnosis service for patients with acute stroke. It has been approved by the Royal Stoke University Hospital Clinical Governance Committee as a service evaluation.

### Setting

The hypnosis service to treat functional stroke was introduced on the acute combined acute hyper-acute stroke unit of the Royal Stoke University Hospital, a comprehensive stroke unit providing acute stroke treatments including thrombolysis and mechanical thrombectomy for 1200 patients per year, in March 2014 and is used in addition to standard care. Standard care for functional strokes includes a full diagnostic work-up, discussion of the results of investigations, an explanation of the functional nature of their symptoms, physiotherapy, occupational therapy, and speech and language therapy, where applicable. If indicated and acceptable, the first hypnosis session is given within 24–48 h of diagnosis of functional stroke, while the patient is still on the acute stroke unit. All patients are followed up after four to six weeks.

### Patients

Adults with a clinical diagnosis of functional stroke were included. The diagnosis was confirmed by an independent stroke physician and supported by normal imaging results. Functional overlay was diagnosed if patients had a clinical or radiological diagnosis of stroke, but symptoms and signs were variable, inconsistent, and out of proportion with the radiological findings. Hypnotherapy was offered to patients with functional stroke symptoms if their symptoms lasted longer than 24 h. Imaging included a computed tomography head scan and magnetic resonance imaging and was performed to rule out pathology which could account for the presenting symptoms. Hypnosis was not offered to patients who were unable to follow the instructions because of cognitive impairment or if they were unable to speak or understand English. The treating physician informed the patient about the hypnotherapy option, and the rationale for the therapy was explained. Verbal consent was taken before initiating hypnotherapy. Patients who declined the offer of hypnotherapy received standard care.

### Treatment

Hypnosis was delivered by a single hypnosis-trained stroke physician (RS). The treatment was conducted in the therapy room of the stroke unit, with a chaperone present. Patients were sitting on a chair or lying on a bed during hypnosis, depending on ability and preference.

During the first session an in depth history of the presenting symptoms is taken, details of the social and personal background of the patient were explored using a life story format, and the hypnosis procedure was described. Special emphasis was given to explain to the patient that in hypnosis there is no loss of control. Verbal consent for the procedure was taken from each patient.

Hypnosis was provided using symptom-based and insight-based approaches either in isolation or in combination, subject to patient history. The symptom-based approach uses mental imagery including normal function of the affected body part in a deep hypnotic state to addresses symptoms directly. This was used as the primary approach in all patients. The insight-based approach was employed if the patient did not respond to the symptom-based approach alone, and underlying psychological trauma was suspected as a potential cause. Use of this approach usually required a further treatment session, informed by a more detailed examination of comorbidities (Appendix 4) and involved various techniques such as “healing the inner child,”^12^ desensitization, neurolinguistic programming,^13^ circle therapy,^14^ and cognitive behavioral therapy.

Hypnosis was conducted in five successive steps: relaxation, induction, deepening, suggestion, and reversal. Relaxation was facilitated by a quiet undisturbed environment and an unchallenging conversation aimed at putting the patient at rest. Induction used standard techniques such as hand-raising, finger-spreading, or eye fascination, which capture the patient’s attention without being challenging. Deepening was attained using imagery such as going down a staircase, reaching a golden color bridge, observing a candle flame and stimulating several auditory, visual, sensory, smell and taste perceptions via suggestion. This step varies in duration, but usually takes longer than steps 1 and 2. Depth of hypnosis was assessed using the six-stage Arons Depth Scale (Appendix 2), where 1 represents a light state of hypnosis (like falling asleep) and 6 represents deep hypnosis (profound somnambulism).^11^ Once adequate depth (stage 2 or higher) was attained, suggestion was used to achieve the therapeutic goal. Different imagery, tactile auditory, and sensory suggestions were employed to visualize normal use of the affected limbs, normal sensation, or normal speech (with variations depending on the presenting symptoms) within the context of their usual interactions with family, friends, work, and leisure activities. These suggestions were repeated three to five times, depending on patient response. At the end of the therapy, the patient was brought out of the hypnotic state by counting up from 0 to 5 and was reexamined to document whether there were any changes in, or resolution of, the symptoms. Each treatment session lasted 60 to 90 min. Patients were also taught self-hypnosis to facilitate continued practice at home. This was based on the content and techniques used in the treatment session and supported by an instruction leaflet (Appendix 3) and, where feasible and acceptable, an audio recording of their treatment session.

Appendix 5 summarizes the treatment plan. The number of sessions of hypnotherapy conducted for each patient was determined by the response to treatment with a maximum of 10 weekly appointments. All patients were seen for at least one further session. During this session, a more detailed personal and psychological history was taken. Patients who responded to the first session received further guidance, reinforcement, and, if necessary, insight-based therapy. Patients who had not responded to the first session had further symptom based or insight-based sessions dependent on response and the personal and psychological history. All patients were followed up in a face-to-face consultation at six weeks posttreatment. A second follow-up was conducted at six months via the phone. After this, patients were offered open long-term follow-up for recurrence of the symptoms.

### Assessments

Each patient had a full neurological examination with a record of severity of neurological symptoms using the National Institutes for Health Stroke Scale (NIHSS, scale 0–42) score and disability using the modified Rankin Scale (mRS, 0–6) by a certified professional at baseline. Comorbidities were assessed informally using a life story approach in the first session and more formally using a framework of domains in the follow-up session.^15^

### Definition of treatment response

A patient was considered a responder if there was a reduction of 4 points or more in the NIHSS score, provided the baseline NIHSS score was no less than four. For patients whose baseline NIHSS score was less than 4, a positive response was defined by a fall of the posttreatment NIHSS to zero. Nonresponders were offered further treatment sessions unless they declined.

### Statistical methodology

We developed predictive models for treatment outcomes posthypnosis based on 68 patients and 10 variables (age, sex, suggestibility, functional overlay, hypnotized, satisfactory depth of hypnosis, treatment within seven days of symptom onset, baseline mRS, psychological link, and NIHSS prehypnotherapy, but after at least one session of physiotherapy) detailed in Table S1 (Appendix 1).

*Model 1*: Prediction of the mRS score posthypnotherapy.

We treated the mRS score posthypnotherapy as a continuous variable due to an insufficient number of observations (only 4) in the mRS score 3 category.^16^

*Model 2*: Prediction of the NIHSS score posthypnotherapy.

We treated the NIHSS score posthypnotherapy as a continuous variable in regression analysis since it takes all integer values between 0 and 42 (43 categories).^17^

Both models were built in R statistical software tool (R Core Team, 2018) by means of R-package CARRoT^16,18–21^ which combines principles of good practice from machine learning, such as cross-validation^22^ and those in medical statistics, such as best subset regression,^23^ restricted by the 10 events per variable rule (“one in ten rule”).^24^ The accuracy of the models was tested by running 1000 cross-validations each of which was performed by splitting the data set into training (90%) and test sets (10%) which amounts to 6800 test points for each feasible model.

### Cost-effectiveness

Cost-effectiveness of the intervention was assessed by associating a cost with each mRS status. Costs are based on Dawson et al.^25^ and are shown in Table S5 (Appendix 1). Bed time costs for each relevant mRS category are reported in terms of 95% confidence intervals, the midpoint of which is used as an approximation of the associated bed time. All costs are in Great Britain (GB) pounds. It is assumed that the treatment resulted in no savings if the patient had the same mRS score both prior and posthypnotherapy.

## Results

### Baseline demographic and clinical characteristics

**Figure 1.**
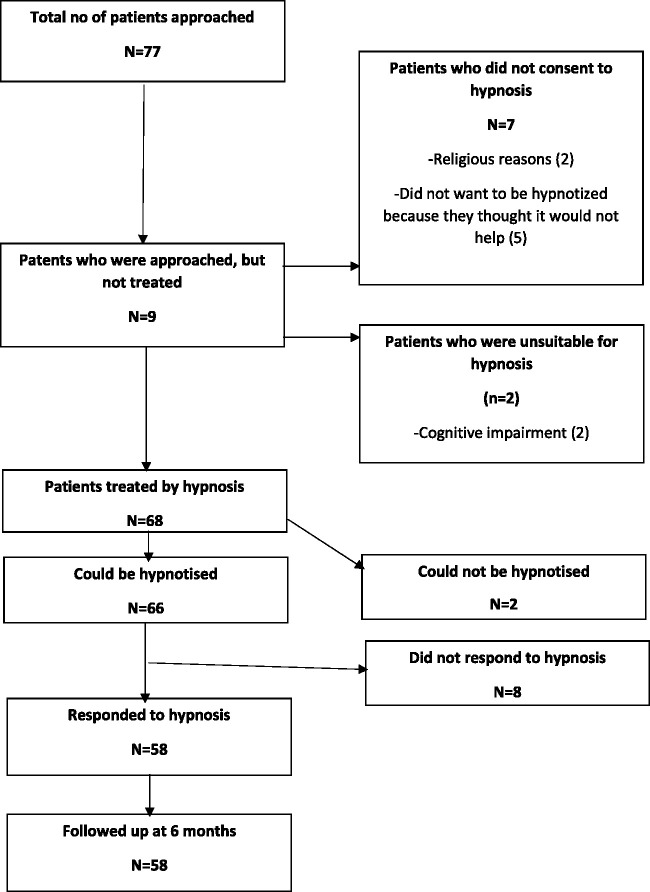
Patient flow.

Seventy-seven patients were approached for potential hypnotherapy. Of these, seven did not consent and two were unsuitable for hypnosis because of cognitive problems. Sixty-eight patients were treated with hypnotherapy between March 2014 and February 2018 and are included in this study (Figure 1).

The mean age of patients with FND was 36.4 years (range 19–76 years) and the majority (n = 52, 76%) were females. The mean NIHSS score was 5.0 (range 1–9). Most (n = 55, 81%) were diagnosed as FND during their first hospitalization with suspected stroke. Hypnotherapy was initiated within seven days of presentation in 56 (82%). The majority (n = 58, 85%) had functional disorder, with the remaining 10 (15%) diagnosed as functional overlay. Patients with functional disorder were younger (mean 33.2; range 19–49 years) than those with functional overlay (mean 54.9; range 37–76 years), and had more severe neurological symptoms (mean NIHSS 5.2 vs. 3.8), but the proportion of females (n = 45, 78% vs. n = 7, 70%) was similar in both groups. Summary results and categories are shown in Table S1 (Appendix 1).

### Symptomatology

Fifty-eight out of 68 patients (85%) presented with limb weakness (right hemiparesis in 36 and left hemiparesis in 22). One patient (1%) had myotonic symptoms in addition to hemiparesis. The initial presentation in four patients (6%) was severe dysarthria and dysfluency syndrome. Three patients (4%) had ataxia, which involved the trunk and the limbs. Three patients (4%) had a loss of sensation in the right side of the body, including the face. Five patients (7%) presented with visual impairments. The pattern of visual deficits included homonymous hemianopia, reduced peripheral fields, reduced acuity, and blurred vision. Two patients (3%) presented primarily with the visual deficit, whereas the others had other neurological deficits in addition to visual symptoms.

### Comorbidities

Of the 68 patients, 55 (81%) had a demonstrable psychological event and/or a psychiatric history. Of these, 41 had a psychological event which could potentially explain the physical symptoms, and in 14 there was no clear link. A potential psychological link was only found in patients with functional disorder (41/58, 71%), but in none of the 10 patients with functional overlay. Possible psychological triggers included childhood trauma (physical, psychological, and sexual), loss of family members, breakdown of a relationship, change in financial circumstances, and taking the role of carers. Most of the events happened either in childhood or a few months prior to presentation with functional disorders.

### Type of hypnosis delivered

Patients were hypnotized based on the nature of their suggestibility. Thirty-four of the 68 patients (50%) were physically suggestible, 20 (29%) were emotionally suggestible, and 14 (21%) were somnambulistic.

### Outcomes

Two out of sixty-eight patients (3%) could not be hypnotized due to severe pain and severe anxiety, respectively. The remaining 66 (97%) were hypnotized successfully. Fifty-eight patients (88% of hypnotized individuals and 85% of all patients treated by hypnosis) responded to the treatment.

**Figure 2.**
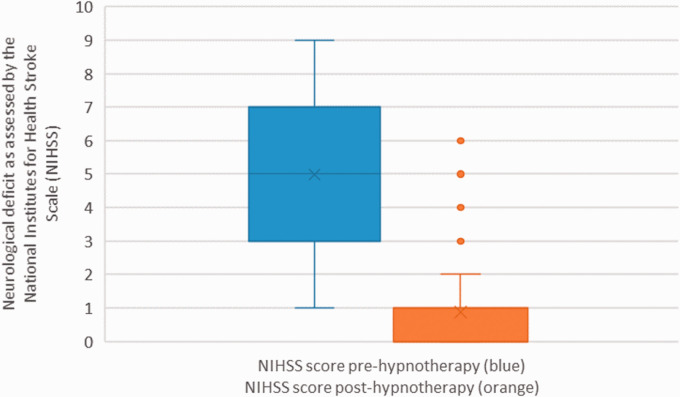
Neurological deficit before and after hypnotherapy. The box plot on the left represents the NIHSS before and the plot on the right the NIHSS after treatment. Lower and upper lines present maxima and minima excluding outliers, middle line is the sample median while the two horizontal lines stand for the first and third quartile of the sample. Outliers are marked with individual points.

**Figure 3.**
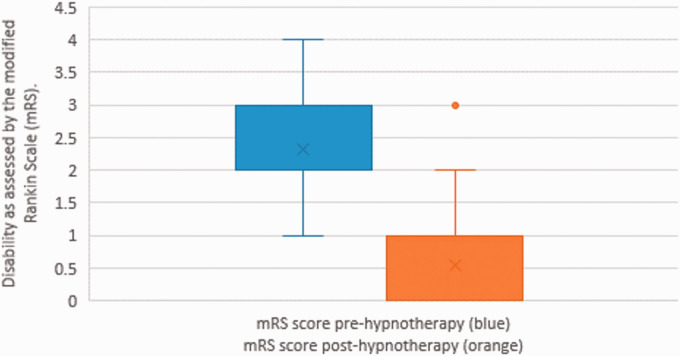
Disability before and after hypnotherapy. The box plot on the left represents the mRS before and the plot on the right the mRS after treatment. Lower and upper lines present maxima and minima excluding outliers, middle line is the sample median while the two horizontal lines stand for the first and third quartile of the sample. Outliers are marked with individual points.

There was a significant improvement in neurological deficits and disability as shown by a reduction in NIHSS (Figure 2) and mRS (Figure 3) scores posttreatment (*p* < 0.0001 for both, *t* test). The mean reduction in NIHSS and mRS scores between baseline and posttreatment was 4.1 and 1.8, respectively. Forty-five patients (66%) had no remaining symptoms (mRS = 0) posttreatment. A full breakdown of pretreatment and posttreatment mRS scores is shown in Table S4 (Appendix 1). There was no sex difference in response to treatment (Tables S7 and S8, Appendix 1).^25^ The mean number of sessions required to achieve symptom control was 1.38 standard deviation (SD) 0.57 and the mean number of sessions required to control the symptoms and treat the underlying psychological trauma was 2.31 SD 1.78. All 68 patients were reviewed in person at six weeks. &The mean NIHSS and mRS were 0.82 SD 1.67 and 0.49 SD 0.92, respectively. There were few symptom changes between posttreatment and six weeks. The NIHSS was unchanged in 56 (82%), improved by 1 point in 8 (12%), and deteriorated in 4 (6%, by 1,1,1,2 points, respectively). The mRS remained the same in 62 (91%), improved by 1 point in 5 (7%), and deteriorated by 1 point in 1 (1%).

All 58 responders could be contacted by phone at six months. Of these, 50 (86%) remained well without recurrence of symptoms. The remaining eight (14%) developed recurrent neurological symptoms which required further hypnotherapy sessions. All responders reported increased levels of motivation and general well-being and were happy to recommend hypnotherapy to others. The patients who did not respond to therapy reported no change in general well-being or motivation.

*Model 1:* Prediction of the mRS score posthypnotherapy.

The model with the smallest average absolute error of 0.24 included age, whether patient was hypnotized or not (reference category “Yes”), whether the depth of hypnosis reached satisfactory levels (reference category “Yes”), mRS score before treatment and NIHSS score prehypnotherapy but after at least one session of physiotherapy. Treatment response (lower mRS score posthypnotherapy) could be predicted by higher age, successful hypnosis, achievement of satisfactory depth of hypnosis, and lower baseline NIHSS and mRS. A detailed breakdown of regression coefficients and their confidence intervals is shown in Table S2 (Appendix 1).

*Model 2*: Prediction of the NIHSS score posthypnotherapy.

The model with the smallest average absolute error of 0.41 included suggestibility (reference categories “physically suggestible” and “somnambulistic”), whether depth of hypnosis reached satisfactory levels (reference category “Yes”), mRS score before treatment and NIHSS score prehypnotherapy but after at least one session of physiotherapy. Treatment response (lower NIHSS) is predicted by physical or somnambulistic suggestibility, achievement of a satisfactory depth of hypnosis and lower baseline NIHSS and mRS. Detailed breakdown of regression coefficients and their confidence intervals can be found in Table S3 (Appendix 1).

#### Cost-effectiveness analysis

Table S4 (Appendix 1) shows a breakdown of mRS score changes posthypnotherapy. The majority of transitions (44/68; 65%) were from mild-to-moderate disability (mRS 1–3) to no disability (mRS 0). Most of the others (12) transitioned from moderate to minor disability (mRS 3–1). One transition was very large from mRS 4 to 1. There was no mRS improvement in 8/68 patients (12%). There were no cases where disability increased posthypnotherapy. Using the known costs of each mRS level (Table S5, Appendix 1) health-care savings associated these mRS transitions were calculated. Table S6 (Appendix 1) shows the number of patients who changed mRS levels and the projected cost savings for each transition. The calculated savings for the whole cohort were £204,563. Most savings (£143,382) were made in the group who transitioned form mild-to-moderate to no disability.

The total savings (£204,563/68 patients) translate into £3,008 per patient treated. Since the intervention cost is no more than 1,350 pounds per person (maximally six sessions, with cost of 225 pounds per session), it is cost-effective with a net saving of more than £1,658 pounds (£3,008–£1,350) per patient to the NHS.

## Discussion

This is the first report of the outcomes of introducing hypnosis as a treatment alternative for patients with FND presenting as acute stroke. The majority of patients who were offered the treatment agreed to be treated and 85% responded with complete or almost complete resolution of symptoms with significant reductions in their NIHSS and mRS scores between baseline and the end of treatment. This was maintained over six months with self-reported improvement in general well-being and increased motivation. Most (81%) only needed one session of hypnotherapy for symptomatic improvement. We also built robust, rigorously cross-validated models for predicting response to hypnotherapy. Good performance of these models is demonstrated by low average absolute errors (0.24 and 0.41) for the mRS and NIHSS scores, respectively, providing us with solid understanding of the expected treatment outcome before it commences. We demonstrated the cost-effectiveness of the intervention based on bed time reduction. The treatment is safe since none of the patients exhibited either an adverse effect directly caused by hypnotherapy or an increase in mRS or NIHSS scores after treatment.

Hypnotherapy has been used successfully in the treatment of functional disorders such as irritable bowel syndrome.^26^ There is evidence of its use in the treatment of several other disorders including fibromyalgia,^27^ insomnia,^28^ and posttraumatic stress disorder.^29^ Two randomized controlled studies of hypnotherapy in patients with long-standing FND with stroke like symptoms and other manifestations of conversion disorder reported no benefit and significant improvements with hypnosis respectively.^9,10^ The observed differences between the studies may have been due to the nature of the control group with a waiting list control in the study which showed effectiveness^9^ and additional active treatment in both groups in the negative study.^10^ In our study, patients were not randomized, so there was no control group. The response to treatment was rapid and could be observed immediately after the end of the session. This suggests a causal relation to the intervention. However, as there was no control treatment, it is uncertain whether the observed response was due to the personal interaction between the hypnotist and the patient or a specific effect of hypnosis itself.

FND is a chronic condition and generally requires multiple treatment sessions and involvement of the multidisciplinary team. Both studies of hypnosis in FND included eight or more treatment sessions.^9,10^ In our study, most patients required only a single treatment for symptomatic improvement. This difference may be due to the nature of the underlying disease. Specific treatment for FND is usually only started when the condition has not resolved with simple supportive approaches. In this study, symptoms were acute, and treatment was given within days of onset. This may explain the high and apparently sustained response rate.

Clinical characteristics of the patients included on our study were similar to those described in other studies with a high proportion of younger females.^3,9,10,30^ In this study, we found that characteristics of patients with functional symptoms differed from those with functional overlay, with the overlay group being older (mean 55 vs. 33 years) and less severe (mean NIHSS 3.8 vs 5.2). A psychological event was documented in a significant number of patients with functional disorders, whereas this was not evident in patients with functional overlay.

A key element of the type of hypnotherapy employed in this study is imagery. This was used in the induction, deepening, for therapeutic suggestions, and for reversal of hypnosis. Mental imagery without hypnosis^31^ is a therapeutic modality of its own and has been used for the treatment of the treatment various conditions including central neuropathic pain^32^ and depression.^33^ As imagery was part of a complex intervention in this study, it is unclear how much this aspect contributed to treatment effect.

Our results show that hypnotherapy could potentially save more than £1,600 per patient on average. If effectiveness is confirmed and the treatment were to be applied widely hypnotherapy could potentially save millions of pounds annually to the NHS. Wider implementation would require clear specification of hypnotic techniques, demonstration of interoperator reproducibility and training of staff in hypnosis techniques.

A limitation of this study is that it is observational. We can therefore not exclude that the improvement in symptoms was due to the natural course of the illness rather than the intervention itself. Furthermore, it is a single center study, and treatment was delivered by a single hypnotist. A multicenter randomized clinical trial is needed to validate the effectiveness of this novel treatment rigorously. Assessment of psychological events and psychiatric comorbidities was done primarily to inform the therapeutic approach, rather for formal identification of psychological or psychiatric trauma and may have over or underestimated truly causative events. Calculation of the costs of stroke was based on published reference values for different levels of disability. These may be higher now than at the time of publication.

To the best of our knowledge, this is the largest study of the use of hypnosis for the treatment of FND presenting as acute stroke in routine clinical practice. In this patient group, hypnotherapy was associated with rapid and sustained recovery of symptoms. These findings need to be confined in a prospective randomized controlled study with a wider range of operators providing the treatment.

## Supplemental Material

sj-pdf-1-wso-10.1177_1747493021995590 - Supplemental material for The use of hypnotherapy as treatment for functional stroke: A case series from a single center in the UK Click here for additional data file. Supplemental material, sj-pdf-1-wso-10.1177_1747493021995590 for The use of hypnotherapy as treatment for functional stroke: A case series from a single center in the UK by Ranjan Sanyal, Marko Raseta, Indira Natarajan and Christine Roffe in International Journal of Stroke

sj-pdf-2-wso-10.1177_1747493021995590 - Supplemental material for The use of hypnotherapy as treatment for functional stroke: A case series from a single center in the UK Click here for additional data file. Supplemental material, sj-pdf-2-wso-10.1177_1747493021995590 for The use of hypnotherapy as treatment for functional stroke: A case series from a single center in the UK by Ranjan Sanyal, Marko Raseta, Indira Natarajan and Christine Roffe in International Journal of Stroke

sj-pdf-3-wso-10.1177_1747493021995590 - Supplemental material for The use of hypnotherapy as treatment for functional stroke: A case series from a single center in the UK Click here for additional data file. Supplemental material, sj-pdf-3-wso-10.1177_1747493021995590 for The use of hypnotherapy as treatment for functional stroke: A case series from a single center in the UK by Ranjan Sanyal, Marko Raseta, Indira Natarajan and Christine Roffe in International Journal of Stroke

sj-pdf-4-wso-10.1177_1747493021995590 - Supplemental material for The use of hypnotherapy as treatment for functional stroke: A case series from a single center in the UK Click here for additional data file. Supplemental material, sj-pdf-4-wso-10.1177_1747493021995590 for The use of hypnotherapy as treatment for functional stroke: A case series from a single center in the UK by Ranjan Sanyal, Marko Raseta, Indira Natarajan and Christine Roffe in International Journal of Stroke

sj-pdf-5-wso-10.1177_1747493021995590 - Supplemental material for The use of hypnotherapy as treatment for functional stroke: A case series from a single center in the UK Click here for additional data file. Supplemental material, sj-pdf-5-wso-10.1177_1747493021995590 for The use of hypnotherapy as treatment for functional stroke: A case series from a single center in the UK by Ranjan Sanyal, Marko Raseta, Indira Natarajan and Christine Roffe in International Journal of Stroke

## References

[1] Carson A Ringbauer R Stone J McKenzie L Warlow C Sharpe M . Do medically unexplained symptoms matter? A prospective cohort study of 300 new referrals to neurology outpatient clinics. J Neurol Neurosurg Psychiatry 2000; 68: 207–210. 10644789 10.1136/jnnp.68.2.207 PMC1736779

[2] Nimnuan C Hotopf M Wessely S . Medically unexplained symptoms: an epidemiological study in seven specialities. Psychosom Res 2001; 51: 361–367. 10.1016/s0022-3999(01)00223-9 11448704

[3] Snijders T de Leeuw F Klumpers U Kappelle L van Gijn J . Prevalence and predictors of unexplained neurological symptoms in an academic neurology outpatient clinic – an observational study. J Neurol Neurosurg Psychiatry 2004; 251: 66–71. 10.1007/s00415-004-0273-y 14999491

[4] Fink P Hansen M Søndergaard L . Somatoform disorders among first-time referrals to a neurology service. Psychosomatics 2005; 46: 540–548. 16288133 10.1176/appi.psy.46.6.540

[5] Healthcare Improvement Scotland. *Stepped care for functional neurological symptoms*, 2012, NHS Scoland.

[6] O’ Neal M Baslet G . Treatment for Patients with a functional neurological disorder (conversion disorder): an integrated approach. Am J Psychiatry 2018; 175: 307–314. 29606068 10.1176/appi.ajp.2017.17040450

[7] Department of Health England. *The economic case for improving efficiency and quality in mental health* 2011. gov.uk.

[8] Moene F and Roelofs K. Hypnosis in the treatment of conversion and somatization disorders. In: Nash M and Barnier A (eds) *The Oxford handbook of hypnosis: theory, research, and practice*. Oxford: Oxford University Press, 2008, pp. 625–646.

[9] Moene F Spinhoven P Hoogduin K van Dyck R . A randomised controlled clinical trial on the additional effect of hypnosis in a comprehensive treatment programme for inpatients with conversion disorder of the motor type. Psychother Psychosom 2002; 71: 66–76. 11844942 10.1159/000049348

[10] Moene F Spinhoven P Hoogduin K van Dyck R . A randomized controlled clinical trial of a hypnosis-based treatment for patients with conversion disorder, motor type. Int J Clin Exp Hypn 2003; 51: 29–50. 12825917 10.1076/iceh.51.1.29.14067

[11] Arons H. *Master course in hypnotism*. South Orange: Power Publishers, 1961.

[12] Beveridge K Cheung M . A spiritual framework in incest survivours treatment. J Child Sexual Abuse 2004; 13: 105–120. 10.1300/J070v13n02_06 15388414

[13] Sturt J Ali S Robertson W . Neurolinguistic programming: a systematic review of the effects on health outcomes. Br J Gen Pract 2012; 62: 757–764. 10.3399/bjgp12X658287 PMC3481516 23211179

[14] Rafiq M . Circle therapy for headache management: case studies. Anaestesia Pain Intens Care 2020; 24: 101–104.

[15] Semple D and Symth R. *Oxford handbook of psychiatry*. Oxford University Press, 3rd ed. 2013.

[16] Bazarova A and Raseta M. *CARRoT: Predicting Categorical and Continuous Outcomes Using One in Ten Rule*. R package version 0.1.0. 2020.

[17] Rhemtulla M Brosseau-Liard P Savalei V . When can categorical variables be treated as continuous?: a comparison of robust continuous and categorical SEM estimation methods under suboptimal conditions. Psychol Methods 2012; 17: 354–373. 22799625 10.1037/a0029315

[18] Smith S Bazarova A Ejenavi E , et al. A multicentre development and validation study of a novel lower gastrointestinal bleeding score—the Birmingham score. Int J Colorect Dis 2020; 35: 285–293. 10.1007/s00384-019-03459-z 31845024

[19] Nayak S Wright H Bazarova A Raseta M . A novel tool for the prediction of clinical outcomes following mechanical thrombectomy. Jf Neuro Intervent Surg 2019; 11: A27.1–A27. 10.1016/j.crad.2020.06.026 32718742

[20] Rutter A Crees J Wright H , et al. Identification of a glass substrate to study cells with Fourier Transform Infrared Spectroscopy: are we closer to spectral pathology? Appl Spectrosc 2019; 74: 178–186. 31517513 10.1177/0003702819875828

[21] R Core Team (2019). R: A language and environment for statistical computing. R Foundation for Statistical Computing, Vienna, Austria.

[22] Geisser S. *Predictive inference*. Milton Park: Taylor & Francis, 1993.

[23] Zhang Z . Variable selection with stepwise and best subset approaches. Ann Transl Med 2016; 4–7: 136. 10.21037/atm.2016.03.35 PMC4842399 27162786

[24] Peduzzi P Concato J Kemper E Holford T Feinstein A . A simulation study of the number of events per variable in logistic regression analysis. J Clin Epidemiol 1996; 49: 1373–1379. 8970487 10.1016/s0895-4356(96)00236-3

[25] Dawson J Lees J Chang T , et al. Association between disability measures and healthcare costs after initial treatment for acute stroke. Stroke 2007; 38: 1893–1898. 17446431 10.1161/STROKEAHA.106.472381

[26] Gonsalkorale W Miller V Afzal A Whorwell P . Long term benefits of hypnotherapy for irritable bowel syndrome. Gut 2003; 52: 1623–1629. 14570733 10.1136/gut.52.11.1623 PMC1773844

[27] Picard P Jusseaume C Boutet M Dualé C Mulliez A Aublet-Cuvellier B . Hypnosis for management of fibromyalgia. Int J Clin Exp Hypn 2013; 61: 111–123. 23153388 10.1080/00207144.2013.729441

[28] Stanton H . Hypnotic relaxation and the reduction of sleep onset insomnia. Int J Psychosom 1989; 36: 64–68. 2689375

[29] Spiegel D Cardeña E . New uses of hypnosis in the treatment of posttraumatic stress disorder. J Clin Psychiatry 1990; 51: 39–43. 2211565

[30] Gargalas S Weeks R Khan-Bourne N , et al. Incidence and outcome of functional stroke mimics admitted to a hyperacute stroke unit. J Neurol Neurosurg Psychiatry 2017; 88: 2–6. 26319438 10.1136/jnnp-2015-311114

[31] Pearson J Naselaris T Holmes E Kosslyn S . Mental imagery: functional mechanisms and clinical applications. Trends Cogn Sci 2015; 19: 590–602. 26412097 10.1016/j.tics.2015.08.003 PMC4595480

[32] Kaur J Ghosh S Sahani A Sinha JK . Mental imagery training for treatment of central neuropathic pain: a narrative review. Acta Neurologica Belgica 2019; 119: 175–186. 30989503 10.1007/s13760-019-01139-x

[33] Blackwell S Browning M Mathews A , et al. Positive imagery-based cognitive bias modification as a web-based treatment tool for depressed adults: a randomized control trial. Clin Psychol Sci 2015; 3: 91–111. 25984421 10.1177/2167702614560746 PMC4359210

