# Supplementary material for: The use of hypnotherapy as treatment for functional stroke: A case series from a single center in the UK
Source: Int J Stroke. 2021 Feb 27;17(1):59–66. doi: 10.1177/1747493021995590 (PMC8739735; doi:10.1177/1747493021995590)
Supplement: sj-pdf-1-wso-10.1177_1747493021995590 - Supplemental material for The use of hypnotherapy as treatment for functional stroke: A case series from a single center in the UK [file sj-pdf-1-wso-10.1177_1747493021995590.pdf]

Appendix 1

Table S1: Summary of baseline variables

Table S2 and Table S3: Best performing models specification

Table S4: Summary of functional improvement post-hypnotherapy

Table S5: Proxy cost of stroke as measured by bed time alone stratified via mRS score

Table S6: Cost reduction post-hypnotherapy as stratified by the reduction in mRS score

Table S7: Breakdown of treatment effectiveness as measured by reduction in the NIHSS score

Table S8: Breakdown of treatment effectiveness as measured by reduction in the mRS score

Table S1 Summary of baseline variables

Variable	Type	Categories	Data<br>N=68
Age (years)	Continuous	NA	Mean 36.4 SD 11.9
Gender	Binary	Male or Female	Female N=52 (76%)
Suggestibility	Categorical	Physically suggestible, Emotionally suggestible or Somnambulistic	Physically suggestible N=34 (50%)<br>Emotionally suggestible N=20 (29%)<br>Somnambulistic N=14 (21%)
Functional Overlay	Binary	Yes or No	Yes N=10 (15%)
Hypnotised	Binary	Yes or No	Yes N=66 (97%)
Depth of hypnosis reached satisfactory level	Binary	Yes or No	Yes N=57(84%)
Treatment within 7 days of onset of symptoms	Binary	Yes or No	Yes N=56 (82%)
Baseline mRS	Ordinal numerical	Scale 0-6	Mean 2 Range 1-9
Psychological link established	Binary	Yes or no	Yes N=56 (82%)
NIHSS pre-hypnosis but after ≥1 session of physiotherapy	Ordinal numerical	Scale 0-35	Mean 5 Range 1-4

NIHSS: National Institutes for Health Stroke Scale, mRS: modified Rankin Scale, N/A not applicable

Table S2 Model 1: Prediction of mRS post-hypnotherapy

	Parameter Estimate	95 % CI for the parameter estimate
Age	-0.011	(0.021,-0.0014)
Hypnotized (reference category ‘’yes’’)	-0.491	(-1.241,0.259)
Depth of hypnosis reached satisfactory level (reference category ‘’yes’’)	-1.739	(-2.084,-1.349)
mRS before treatment	0.286	(0.055,0.518)
NIHSS before hypnotherapy but after at least one session of physiotherapy	0.044	(-0.034,0.121)

Specification of the predictive model with highest accuracy for predicting the exact mRS score post-hypnotherapy. NIHSS: National Institutes for Health Stroke Scale, mRS: modified Rankin Scale

Table S3 Model 2: Prediction of NIHSS score post-hypnotherapy

	Parameter Estimate	95 % CI for parameter estimate
Suggestibility (reference category ''physically suggestible'')	-1.423	(-1.9,-0.946)
Suggestibility (reference category ''somnambulistic'')	-2.530	(-3.234,-1.826)
Depth of hypnosis reached satisfactory levels (reference category ''yes'')	-1.831	(-2.416,-1.246)
mRS before treatment	0.393	(0.066,0.72)
NIHSS before hypnotherapy but after at least one session of physiotherapy	0.151	(0.225,0.476)

Specification of the predictive model with highest accuracy for predicting the exact NIHSS score post-hypnotherapy. NIHSS: National Institutes for Health Stroke Scale, mRS: modified Rankin Scale

Table S4 Breakdown of the probability distribution of reduction in mRS scores post hypnotherapy for the study population

mRS score changes post-treatment	Number of observations (out of 68)	Conditional Probabilities
1→0	10	10/68
2→0	23	23/68
3→0	11	11/68
4→0	1	1/68
2→1	0	0
3→1	12	12/68
4→1	0	0
3→2	2	2/68
4→2	0	0
4→3	1	1/68
0→0	0	0
1→1	1	1/68
2→2	4	4/68
3→3	3	3/68
4→4	0	0

Table S5 Cost of stroke as measured by bed time alone stratified via mRS score

mRS score	Proxy cost	95% confidence interval for bed time cost
0	2953	(2493,3412)
1	3924	(3369,4478)
2	6396	(5784,7008)
3	7906	(7300,8512)
4	10618	(10095,11141)

mRS: modified Rankin Scale

The numbers here are based on Dawson et al[1]

Table S6 Potential cost saving after hypnotherapy

mRS score changes post-treatment	No of individuals for each mRS level change	Savings in GBP based on estimates by Dawson et al[1] (Table S5)	Projected cost saving for the cohort (GBP)
1→0	10	971	9710
2→0	23	3443	79189
3→0	11	4953	54483
4→0	1	7665	7665
3→1	12	3982	47784
3→2	2	1510	3020
4→3	1	2712	2712
Total	60		204563

NIHSS: National Institutes for Health Stroke Scale, mRS: modified Rankin Scale

Potential cost savings after hypnotherapy stratified by mRS change. Savings are calculated by the difference in mean costs between mRS levels in Table S5. mRS score changes recorded here are based on numbers recorded in Table S4.

Table S7 Neurological deficit before and after treatment by sex

Category	Count (n)	NIHSS score pre treatment Mean (SD)	NIHSS score post treatment Mean (SD)
Male	16	5.69 (2.65)	0.88 (1.59)
Female	52	4.77 (2.22)	0.87 (1.66)
Overall	68	4.99 (2.34)	0.87 (1.63)

NIHSS: National Institutes for Health Stroke Scale

Table S8 Disability before and after treatment by sex

Category	Count (n)	mRS score pre treatment Mean (SD)	mRS score post treatment Mean (SD)
Male	16	2.44 (0.73)	0.57 (0.73)
Female	52	2.27 (0.79)	0.54 (0.94)
Overall	68	2.31 (0.78)	0.54 (0.89)

mRS: modified Rankin Scale

1. Dawson J, Lees J, Chang T and al. e. Association between disability measures and healthcare costs after initial treatment for acute stroke. *Stroke*. 2007; 38: 1893-8.
