# Supplementary material for: The use of hypnotherapy as treatment for functional stroke: A case series from a single center in the UK
Source: Int J Stroke. 2021 Feb 27;17(1):59–66. doi: 10.1177/1747493021995590 (PMC8739735; doi:10.1177/1747493021995590)
Supplement: sj-pdf-2-wso-10.1177_1747493021995590 - Supplemental material for The use of hypnotherapy as treatment for functional stroke: A case series from a single center in the UK [file sj-pdf-2-wso-10.1177_1747493021995590.pdf]

Appendix 2. Assessment of depth of hypnosis

Depth of hypnosis was assessed using the Arons Depth Scale ([11]).

Stage 1: Light state of hypnosis characterized by lethargy, relaxation, and eye catalepsy, like the subject is falling asleep.

Stage 2: In this stage there is arm catalepsy, catalepsy of isolated muscle groups, a heavy or floating feeling may also be experienced.

Stage 3: During this stage profound catalepsy is possible, smell and taste changes, number block (aphasia) and partial amnesia can result.

Stage 4: In this stage, subjects exhibit true amnesia, automatic movements, automatic writing, positive olfactory and gustatory hallucinations, name or address block.

Stage 5: Somnambulism begins in this stage and the subject can experience complete anaesthesia and can lose the ability to feel discomfort or touch completely. Positive Hallucinations of all sensory modalities is possible.

Stage 6: Profound Somnambulism. Subjects can experience negative hallucinations where they don't see or hear things that actually do exist.

The following clinical signs were most useful in assessing the depth of hypnosis:

Stillness

Change of breathing

Pallid / waxen complexion

Postural slumping

REM type eye movements

Eyelid fluttering

Swallowing/gulping

Increased lachrymation

Redness around the eyes

Signs of following the suggestions made
