# Supplementary material for: The use of hypnotherapy as treatment for functional stroke: A case series from a single center in the UK
Source: Int J Stroke. 2021 Feb 27;17(1):59–66. doi: 10.1177/1747493021995590 (PMC8739735; doi:10.1177/1747493021995590)
Supplement: sj-pdf-4-wso-10.1177_1747493021995590 - Supplemental material for The use of hypnotherapy as treatment for functional stroke: A case series from a single center in the UK [file sj-pdf-4-wso-10.1177_1747493021995590.pdf]

Appendix 4. Assessment of comorbidities

Basic information: Name, age, marital status, current occupation, route of referral

Presenting complaint(s)

History of presenting complaint(s)

Past psychiatric and medical history – Detail in chronological order

Drug history

Family history: Family tree detailing name, ages, relationship and illnesses of 1st and 2nd degree relatives. Are there any family illnesses?

Childhood: Were there any problems during their pregnancy or delivery? Did they reach development milestones normally? Was their childhood happy? In what sort of family were they raised?

Education: Primary and secondary schools: Did they attend main stream or specialist school? At what age they left school and with what qualifications? Type of further education and qualifications, attainment

Employment: Chronological list of jobs

Relationships: Sexual orientation, chronological account of major relationships, reasons for their breakdown, are they currently in a relationship? Do they have children from current or previous relationship? Who do the children live with? What relationship the patient have with them?

Forensic: Have they being charged or convicted of any offence? What sentences they have received? Do they have any outstanding charge or convictions?

Social background information: Current occupation. Are they working at the moment? If not, how long they are off work and why? Current/family relationship situation. Alcohol and illicit drug use. Main recreational activities.

This has been modified form the standard history detailed in the Handbook of Psychiatry ([15]).
