# Supplementary figures and images for: The use of hypnotherapy as treatment for functional stroke: A case series from a single center in the UK
Source: Int J Stroke. 2021 Feb 27;17(1):59–66. doi: 10.1177/1747493021995590 (PMC8739735; doi:10.1177/1747493021995590)

**Appendix 5: Treatment plan for hypnosis**

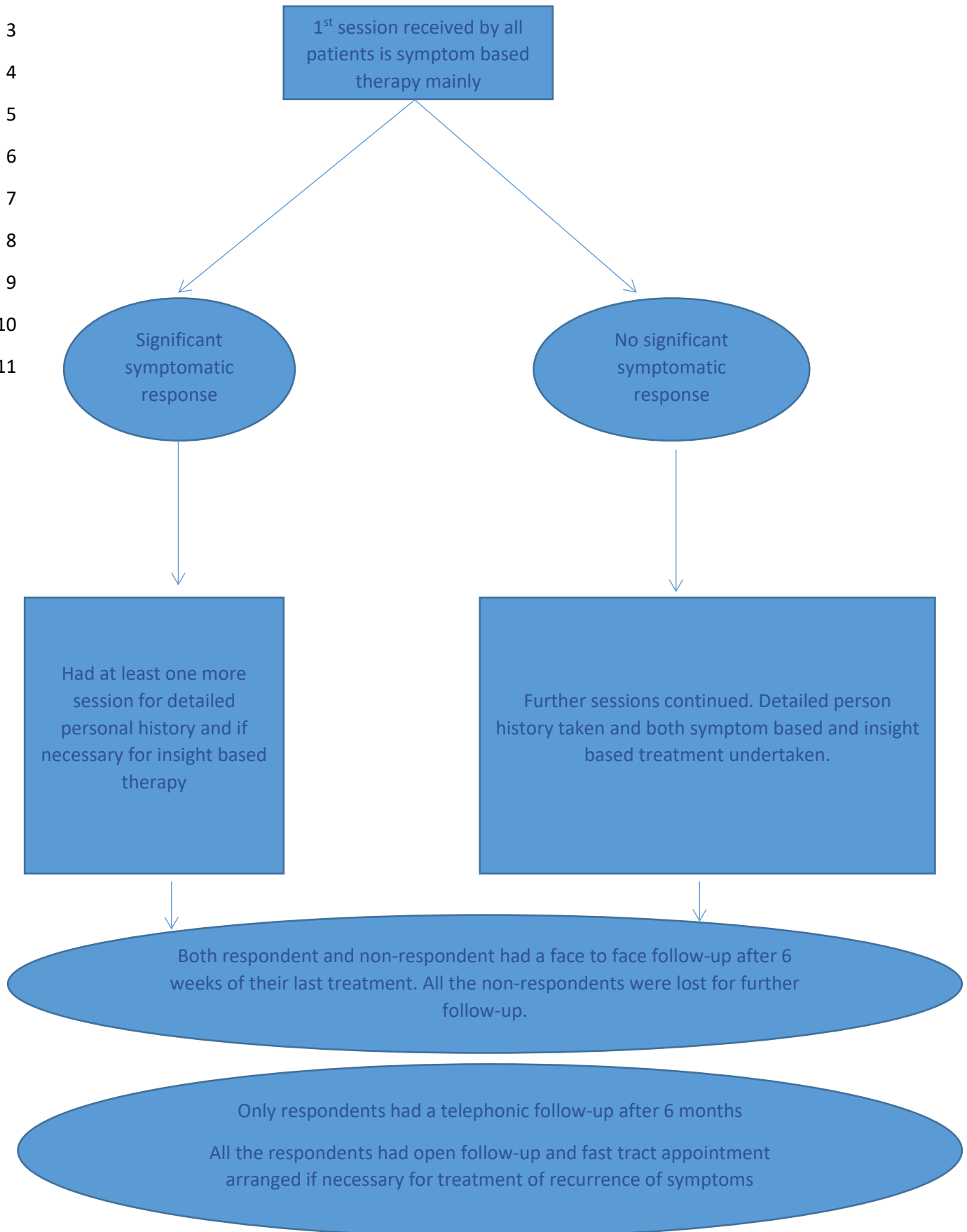

Supplement: sj-pdf-5-wso-10.1177_1747493021995590 - Supplemental material for The use of hypnotherapy as treatment for functional stroke: A case series from a single center in the UK [file sj-pdf-5-wso-10.1177_1747493021995590.pdf]
