# Supplementary material for: The use of hypnotherapy as treatment for functional stroke: A case series from a single center in the UK
Source: Int J Stroke. 2021 Feb 27;17(1):59–66. doi: 10.1177/1747493021995590 (PMC8739735; doi:10.1177/1747493021995590)
Supplement: sj-pdf-3-wso-10.1177_1747493021995590 - Supplemental material for The use of hypnotherapy as treatment for functional stroke: A case series from a single center in the UK [file sj-pdf-3-wso-10.1177_1747493021995590.pdf]

Appendix 3. Patient Instructions for Self-Hypnosis

Self-hypnosis involves five steps outlined below.

Please choose a quiet place where you will not be disturbed for next 15 minutes.

1. Breathing exercise to relax – Close your eyes and take deep breaths in and out for 5 times

2. Imagine or have an idea of light going down your body relaxing each and every tissue in the body from the top of the head to the tip of the toes.

3. Imagine yourself standing on the top of a staircase with golden colour rales and red carpets on this 20 steps stair case. Count 20 to 0 going down the stair case one step at a time.

4. To reach a place of safety and visualise the changes that need to be achieved. To visualise changes up to maximum of three things. Try to repeat the visualisation three times.

5. **Count from 1 to 5 to come out from the state of hypnosis**

Please practise this twice a day.
